# Antin-diabetic cognitive dysfunction effects and underpinning mechanisms of phytogenic bioactive peptides: a review

**DOI:** 10.3389/fnut.2024.1517087

**Published:** 2025-01-10

**Authors:** Xiaoli Liu, Shenglian Mao, Yuxue Yuan, Zilin Wang, Yang Tian, Liang Tao, Jiahe Dai

**Affiliations:** ^1^College of Food Science and Technology, Yunan Agricultural University, Kunming, China; ^2^Engineering Research Center of Development and Utilization of Food and Drug Homologous Resources, Ministry of Education, Yunnan Agricultural University, Kunming, China; ^3^Pu’er College, Pu’er, China

**Keywords:** cognitive improvement, cognitive function, diabetic cognitive dysfunction, diabetic disease, phytogenic bioactive peptides

## Abstract

Diabetic cognitive dysfunction is one of the important comorbidities and complications of diabetes, which is mainly manifested by loss of learning ability and memory, behavioural disorders, and may even develop into dementia. While traditional anti-diabetic medications are effective in improving cognition and memory, long-term use of these medications can be accompanied by undesirable side effects. Therefore, there is an urgent need to find safe and effective alternative therapies. Accumulating evidence suggests that phytogenic bioactive peptides play an important role in the regulation of cognitive dysfunction in diabetes. In this review, we explored the relationship between diabetes mellitus and cognitive dysfunction, and the potential and underlying mechanisms of plant-derived bioactive peptides to improve diabetic cognitive dysfunction. We found that plant-derived active peptides alleviate diabetic cognitive impairment by inhibiting key enzymes (e.g., *α*-glucosidase, α-amylase) to improve blood glucose levels and increase antioxidant activity, modulate inflammatory mediators, and address intestinal dysbiosis. In conclusion, plant-derived active peptides show strong potential to improve diabetic cognitive impairment.

## 1 Introduction

**Figure 1:**
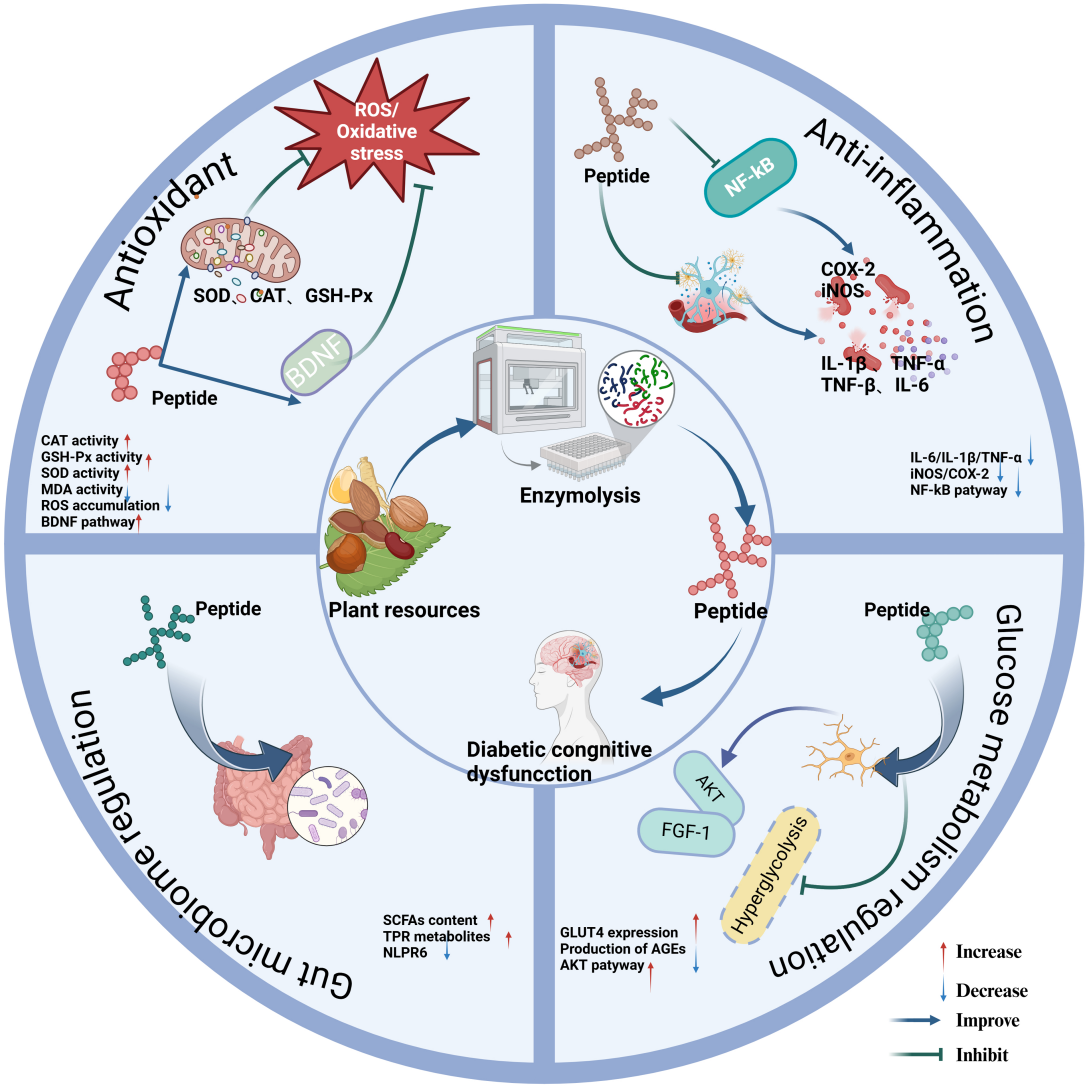
Regulatory mechanism of phytogenic bioactive peptides on diabetic cognitive dysfunction. Created in BioRender.com.

Diabetes mellitus is a metabolic disease characterised by insulin resistance and pancreatic *β*-cell dysfunction leading to disorders in glucose glucose metabolism, which can lead to chronic complications in the body such as cardiovascular disease, nephropathy, retinopathy and others (1). As the impact of diabetes pathogenesis has been intensively studied, there is growing evidence that people with impaired glucose tolerance and diabetes have an increased risk of developing cognitive dysfunction compared to healthy individuals, and a 50% increased risk of dementia in type 2 diabetes (T2DM) (2). The severity of cognitive impairment can significantly affect a patient’s daily life, and the global prevalence of diabetes is increasing year by year with an ageing population, changing dietary patterns, and the accelerated pace of modern life. The World Health organisation (WHO) estimates that approximately 552 million adults are expected to have diabetes by 2030, and diabetes-related cognitive dysfunction is likely to be a major challenge in terms of future health resource requirements (3, 4) (Figure 1).

T2DM is the most common type of diabetes mellitus, and it is widely recognised that insulin resistance (IR) plays a key role in the development of T2DM and its organ-related complications, but the pathogenesis of cognitive dysfunction in diabetes mellitus is not yet fully understood (5). It is known that insulin is involved in neuronal survival, synaptic plasticity, memory, and cognitive function in the central nervous system (CNS), and existing hypotheses suggest that similar to diabetic neuropathy, impaired insulin signalling pathways due to elevated blood glucose and lipids may be the main metabolic mechanism that induces the occurrence of diabetic cognitive dysfunction (6). IR induced metabolic disturbances lead to degradation of brain function, and prolonged uncontrolled high blood glucose levels adversely affect a variety of metabolic pathways such as oxidative stress, formation of advanced glycosylation end products (AGEs), and protein kinase C activation, while metabolic disturbances inducing cerebral microvascular damage affect the extent of cognitive impairment (1, 7). The onset of cognitive dysfunction in diabetes is multifactorial. In addition to genetic factors, lifestyle habits (e.g., diet, sedentariness, and stress), and environmental factors (pesticides, heavy metals, and industrial by-products) have been linked to the progression of cognitive impairment, and these factors may further influence the signalling exchanges between the gut and the brain by affecting the abundance and diversity of gut flora. Traditionally, drugs used for the treatment of neurodegenerative diseases have mainly consisted of acetylcholinesterase inhibitors (ChEIs) and NMDA receptor antagonists (8). Although these drugs are effective in improving cognitive function and daily living abilities, they have limited efficacy in diabetic cognitive dysfunction, and available data suggest that antidiabetic drugs have the potential to improve cognitive dysfunction and dementia (9). The main antidiabetic drugs used clinically are thiazolidinediones (e.g., troglitazone, rosiglitazone), metformin, sulphonylureas and glinides (10). Although these drugs may slow the rate of cognitive decline by lowering blood glucose and enhancing central insulin signalling in the brain, patients can experience adverse effects such as decreased appetite, nausea, abdominal discomfort and diarrhoea while taking them (11). Currently, due to the need for health and safety, dietary control to prevent or influence the development of pathological conditions rather than treatment with drugs after onset may be more consistent with the idea of pursuing a healthy lifestyle. Therefore, it has become a research hotspot to develop foodborne active substances to improve diabetes mellitus and prevent cognitive function decline (12).

A growing body of evidence suggests that dietary nutrients from natural foods have great potential to positively modulate diabetes and its cognitive dysfunction, providing opportunities for safe and cost-effective nutritional modifiers (13). Nutrients such as polyphenols, flavonoids, glycosides, phosphatidylserine, proteins and peptides have been reported to exhibit positive effects in the management of cognitive deficits (14, 15). Among them, several studies have found that bioactive peptides of plant origin not only have the potential to regulate diabetes, but importantly also have neuroprotective and cognitive improvement functions (16, 17). Bioactive peptides isolated from plant proteins, generally consisting of 2–20 amino acids, have attracted widespread attention in the academic and health care communities for their good tissue affinity, specificity, and bioactivities such as antioxidant, anti-inflammatory, and hypoglycemic properties (17, 18). In this paper, we summarise the research progress of phytogenic bioactive peptides in improving diabetes and cognitive dysfunction, and focus on the role of phytogenic peptides in improving diabetes and cognitive dysfunction and its molecular mechanism, aiming to provide systematic theoretical support for the effective implementation of phytogenic bioactive peptides in the intervention of diabetes and cognitive dysfunction.

## 2 Factors influencing diabetes and its associated cognitive impairment

### 2.1 Cerebral microvascular disorder and diabetic cognitive dysfunction

Cerebral microvascular damage may be one of the mechanisms associated with cognitive dysfunction in diabetes (19). The brain performs cognition and modulates cardiovascular homeostasis. As an important channel of essential oxygen and energy sources for the brain, cerebral microvessels are an important component of the blood–brain barrier (BBB), and its disruption is an early pathophysiological mechanism in neurodegenerative diseases (20). BBB is a biological and physical barrier consisting of astrocytes, pericytes, and brain microvascular endothelial cells (BMECs) that maintains the dynamic between the peripheral circulation and the central nervous system (21). The persistent hyperglycaemic state of diabetes leads to the deposition of advanced glycosylation end-products (AEGs) in the vascular wall of diabetic patients. The accumulation of AEGs and their binding to the receptor for glycosylation end-products (RAGE) promotes oxidative stress, which activates the NF-κB signalling pathway, upregulates the expression of target genes and triggers inflammation (22). Excessive production of RAGE on brain microvascular endothelial cells increases Aβ transport into the brain tissue and decreases its removal, leading to the accumulation of Aβ in the brain tissue, which in turn triggers a series of neurodegenerative pathological changes, such as the formation of amyloid plaques, the formation of neurofibrillary tangles, and the loss of neurons (23). Insulin resistance as well as the hypertensive response can cause damage to the structural integrity and transport function of the blood–brain barrier, increase the permeability of the blood–brain barrier (24), and the entry of free fatty acids (FFA) and other plasma components into the brain tissue to disrupt the homeostatic balance of the brain, as well as the entry of immune cells and inflammatory factors in the peripheral circulation into the CNS, which can cause further neuronal damage, thus leading to cognitive dysfunction (19). On this basis, the presence of microvascular alterations in a pre-diabetic mouse model also suggests that early hyperinsulinaemia and insulin resistance are sufficient to induce vascular damage and that blood–brain barrier dysfunction precedes cognitive decline (25). Delaying the onset of diabetes by altering the chronic hyperglycaemic state through plant-derived bioactive peptides may be an effective way to improve the associated cognitive dysfunction.

### 2.2 Neuroinflammation in the brain and cognitive dysfunction in diabetes

Neuroinflammation itself is a defence mechanism against acute the CNS damage and ameliorates the effects of toxic substances produced in brain nerve cells, but persistent neuroinflammation inhibits nerve regeneration, leading to neurodegeneration making the patient cognitively impaired. Neurodegeneration has been observed in animal models of diabetes. Epidemiology suggests that mild cognitive impairment in diabetes is present in all age groups, but predominantly occurs in older adults (> 65 years) (26). Rohden et al. (27) found that the cellular and molecular mechanisms of neuroinflammation in Alzheimer’s disease and diabetic cognitive dysfunction may have relevance, and that the cell types involved in the inflammatory response in CNS are predominantly astrocytes and microglia, and that glia and neurons interact with each other via synapses, neurotransmitters, and chemicals. In the brain, hyperglycaemia activates microglia, and microglia activation and polarisation can mediate central inflammatory responses, neuronal apoptosis and exacerbate central neuropathy leading to dementia (28). Moreover, persistent hyperglycaemia triggers activation of the (NF-κB) pathway and release of pro-inflammatory factors, leading to an imbalance between the pro- and anti-inflammatory networks, resulting in an increase in reactive oxygen species and the production of a large number of inflammatory mediators, which affects the functioning of the mitochondria and leads to neuronal damage and degeneration (29).

Neuroinflammation can damage neuron-associated axons and myelin fractions, Paul et al. (30) suggesting that insulin resistance and glucose toxicity within the CNS of patients with T2D damages axon integrity, inhibits nerve impulses, and degrades myelin in oligodendrocytes, leading to cognitive dysfunction. These inflammatory responses may be an important etiological factor in the development of memory impairment and behavioural changes in the nervous system. Meng et al. (31) showed that neurons in the brains of patients with T2DM secrete the regulatory factor angiopoietin-like protein 8 (ANGPTL8) into the hippocampus, which activates microglia to up-regulate pro-inflammatory factors and axonal damage, leading to cognitive impairment. Co-localisation of amyloid plaques and neuroprogenitor fibre tangles with activated glial cells in animal models and human brain tissue. Several studies have reported pathological astrogliosis and associated neuronal hypotrophic glucose metabolism in patients with neurodegenerative disease and diabetic model animals, showing increased glial fibrillary acidic protein (GFAP) and significant cellular hypertrophy (32), which has been correlated to some extent with the severity of cognitive impairment in patients with Alzheimer’s disease (AD). Under diabetic conditions, disruption of the regulation of microglia activity by hyperglycaemia occurs through a number of mechanisms, including overproduction of reactive oxygen species (ROS) and glycosylation end products (AGEs), and reduced elimination of Aβ. Y. Li et al. (33) demonstrated that activation of microglial NLRP3 inflammatory vesicles by diabetic mice and BV2 cells via the ROS/JNK MAPKs/NF-κB pathway leads to neuroinflammation. It follows that diabetes-related neuroinflammation exacerbates neurodegenerative disease and is an important regulatory mechanism for cognitive dysfunction.

The development of neuroinflammatory processes in the brain involves the activation of microglia and astrocytes, which is usually triggered by tissue damage and Aβ plaque deposition (34). Aβ attracts and activates microglia and astrocytes, leading to the release of pro-inflammatory mediators such as TNFα, IL-6, IL-2, and IL-1β, as well as reactive oxygen and nitrogen species produced by oxidative stress, which ultimately leads to neuronal cell death (35). In turn, these mediators have the ability to damage neurons while promoting Aβ synthesis and further enhancing microglia activation. In addition, it is known that Aβ induces the expression of enzymes such as nitric oxide synthase (NOS) and reactive oxygen species (ROS), which may lead to neighbouring neuronal damage. These pro-inflammatory mediators, along the lines of ROS, also stimulate *γ*-secretase activity and enhance the expression of amyloid precursor protein (APP), which promotes the processing of APP into the amyloid form (36). When neurons are damaged or die, they release immune signalling molecules that can exacerbate the inflammatory response, thereby increasing the neurotoxic effects of inflammation. These neurons also release the glutamate produced into the surrounding area, which may have a detrimental effect on the health of nearby neurons (37). Notably, there is evidence that the pro-inflammatory cytokine IL-1β plays a role in exacerbating tau pathology by accelerating tau phosphorylation (38).

### 2.3 Neurooxidative stress and cognitive dysfunction in diabetes

The brain is the most oxygen-consuming organ in the human body, with the normal human brain accounting for more than 20 to 30 percent of the body’s total oxygen consumption, but the brain is low in antioxidants, making it more susceptible to the effects of oxidative stress (39). When oxidative stress is excessive or prolonged, the free radicals produced can cause lipid peroxidation, protein denaturation, and nucleic acid base damage, and different biomarkers can reflect oxidative damage to various biomolecules. For example, the levels of 4-hydroxy-2-nonenal (4-HNE) and malondialdehyde (MDA) in the brain reflect the extent of lipid peroxidation (40). Similarly, in microglia mitochondria, levels of 8-hydroxy-2-deoxyguanosine (8-OHdG) have been shown to be regarded as a marker of DNA/ RNA oxidation due to the lack of protection of mitochondrial DNA (mtDNA) by histone proteins and its own limited repair capacity (41). Using targeted proteomics, ENPP-2 was increased in cerebrospinal fluid (CSF) of AD patients with amnestic mild cognitive impairment (aMCI), and ENPP-2 directly reflects the brain glucose steady state (42, 43). Hyperglycemia, hyperinsulinemia, or hypoinsulinemia can cause oxidative stress, and reactive oxygen species (ROS) and reactive nitrogen species (RNS) produced by excessive oxidative stress can disrupt the BBB and further affect central nervous system (CNS) function. Studies have shown that oxidative stress promotes brain insulin resistance, and central nervous system (CNS) insulin resistance can affect neuronal development and increase the risk of neurodegenerative diseases (44).

Oxidative stress has a common role and key link in multiple mechanisms of diabetic cognitive dysfunction and neurodegenerative diseases, and is a bridge between different pathogenic mechanisms of diabetes and cognitive dysfunction, as well as an important link in the pathogenesis of diabetic cognitive impairment. Excessive oxidative stress produces ROS and RON, which mainly include superoxide anion (O_2_^−^), hydroxyl radical (^.^OH), H_2_O_2_, nitrogen dioxide (NO₂) and peroxynitrite (ONOO^−^), these deriving mainly from the mitochondrial electron transport chain, changes in metal valence states and enzymatic processes (e.g., MAO-B, NADPH oxidase) (45). Blood glucose levels affecting antioxidant levels of superoxide dismutase and antioxidants such as catalase or glutathione peroxidase for antioxidant defence, Cardoso et al. (46) analysed cerebral cortex and hippocampal mitochondria from hyperglycaemic and recurrently hypoglycaemic animals and found that cerebral cortical mitochondria exhibited high levels of MDA and *α*-tocopherol and increased glutathione disulphide reductase activity. Reduced manganese superoxide dismutase (MnSOD) activity, reduced glutathione to glutathione disulphide (GSH/GSSG) ratios, and an impaired oxidative phosphorylation system were accompanied by an increase in caspase 9 activity in hippocampal homogenates. Numerous studies have shown that neurogenic fibre tangles (NFTs), which are observed in the brains of patients with AD and diabetic encephalopathy, consist of hyperphosphorylated tau (pTau) (47). In the brain, in order to induce tau phosphorylation, oxidative stress can directly interact with protein kinases, particularly glycogen synthase kinase-3 (GSK 3), increasing GSK-3β activity, which subsequently further disrupts its ability to bind to microtubules, accelerating their depolymerisation and interrupting neural signalling (48).

### 2.4 Gut dysbiosis and cognitive impairment in diabetes mellitus

Human gut microbial communities promote host health through reciprocal relationships between them, and gut ecological dysbiosis is characterised by a reduction in microbially dominant strains and inadequate exposure to beneficial substances leading to poor gut microbial colonisation (49). Whereas the microbe-gut-brain axis connects the gut to the brain, and the enteric nervous system (ENS) exists at the interface between the microbiota and the host, the ENS is structurally and neurochemically similar to the CNS, and thus the pathogenic mechanisms that give rise to ENS disorders may also lead to CNS dysfunction and the nerves connecting the ENS to the CNS may act as a conduit for disease transmission (50). Thus, the ENS is regarded as a second brain that can directly or indirectly respond to the microbiota and its metabolites influencing cognitive functions through gut bacteria (51). The study found that the intestinal microbial imbalance in T2D was characterised by a decrease in the abundance of Bifidobacteria, Bacteroides, Faecalis, Akkermansia and Byrysia ross, while an increase in Rumen coccus, Fusobacterium and Blautella (52). One of the significant changes was a decrease in the number of Gram-positive organisms (53). Increased abundance of Gram-negative microorganisms leads to increased release of lipopolysaccharide (LPS), which disrupts the integrity of the intestinal mucosal barrier and triggers neuroinflammation and neuronal death through a series of steps (54). In addition to this, the brain receives information from the gut through a continuous flow of microbial, endocrine, metabolic and immune factors. This is also considered one of the main factors that promote obesity, diabetes and neuropsychiatric disorders (55).

**Figure 2:**
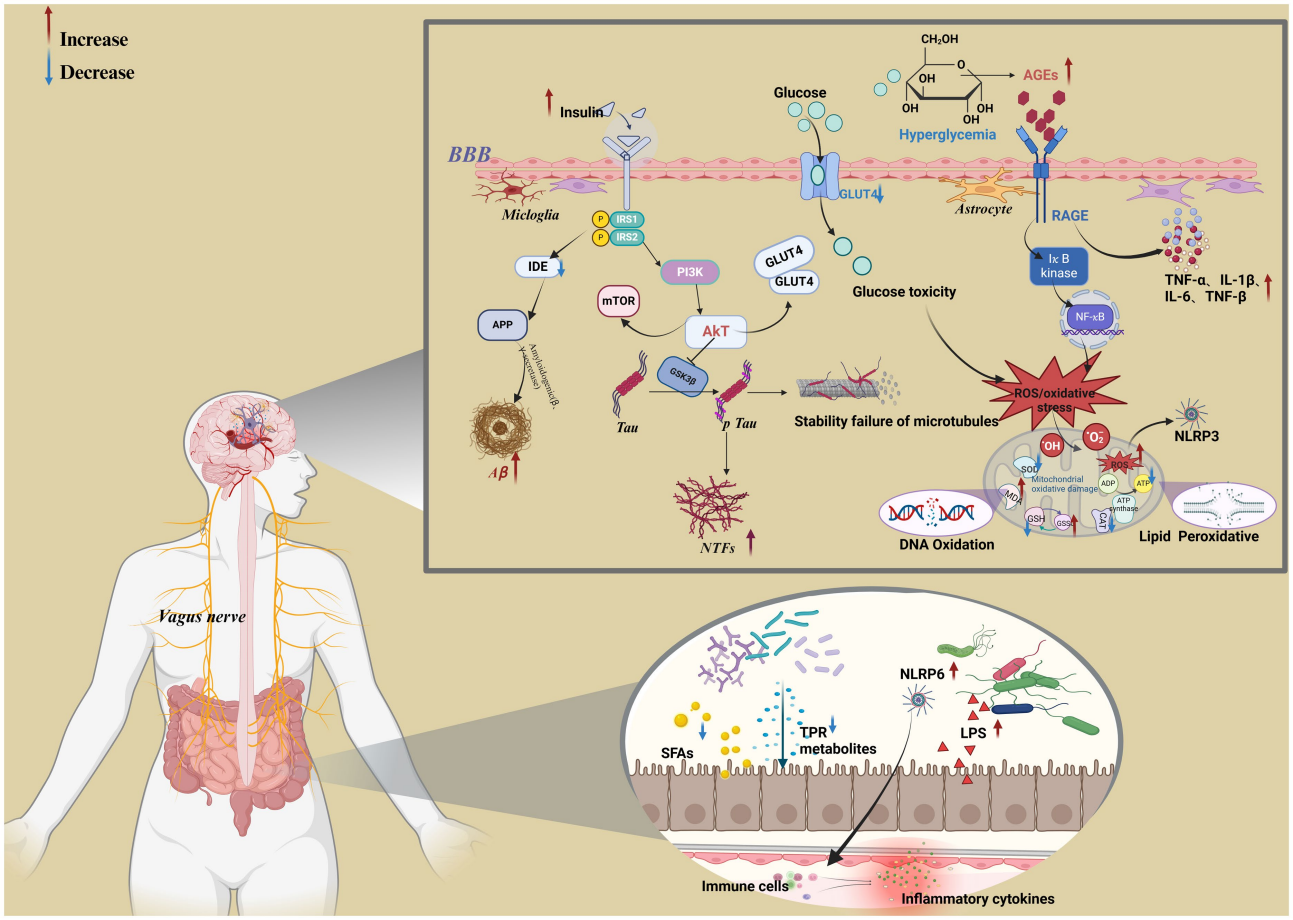
Metabolic mechanisms associated with cognitive dysfunction in diabetes. Created in BioRender.com.

Studies have shown a complex interaction between the gut and the brain, and that gut ecological dysregulation disrupts nervous system homeostasis in two main ways. On the one hand are microflora-associated metabolites such as short-chain fatty acids, tryptophan metabolites, immunostimulants and endogenous cannabinoids that may play a mediating role. On the other hand signalling molecules that operate mainly in the brain, in particular neuropeptide Y, brain-derived neurotrophic factor and *γ*-aminobutyric acid, which are disturbed by microbiological, obesity and diabetes, and are associated with psychiatric disorders (56, 57). The enteric nervous system transmits information from the gut to the brain via information carriers such as vagal afferent neurons, spinal afferent neurons, immune mediators and gut hormones. The influence of the gut microbiota is attributed to the host during disease, ageing, and lifestyle habits. High-sugar, high-fat (HSHF) diets significantly lead to an imbalance in the ratio of bacteria in the phylum Acidobacteria, Firmicutes and Thick-walled bacteria to those in the phylum Anaplasma, Aspergillus, Cyanobacteria, and Actinobacteria, which impairs the gut barrier function, and activates microglial cells in the brain, generating inflammation and affecting the cholinergic system to increase the risk of cognitive dysfunction (58, 59). Transplantation of faecal microorganisms (FMT) from healthy mice into male mice placed on a high-fat diet (HFD) can restore bacterial diversity, improves metabolism associated with poor dietary patterns and reduces hippocampal astrocyte hyperplasia, supporting the role of ecological dysregulation in mediating obesity-induced cognitive impairment (60). Moreover, microbial changes affect gastrointestinal (GIT) dysbiosis, which contributes to the gut-brain axis by increasing systemic LPS, reactive oxygen species, and inflammation. It has been reported that inflammation induced by direct injection of LPS can disrupt BBB and increase vascular permeability, increasing the infiltration of inflammatory mediators, leading to neuroinflammatory response and cognitive dysfunction (61) (Figure 2).

## 3 The role and regulatory mechanism of plant-derived bioactive peptides in the regulation of diabetic cognitive impairment

### 3.1 As a carbohydrate-digesting enzyme inhibitor to regulate diabetic cognitive impairment

A key aspect of diabetes management is the inhibition of enzymes involved in carbohydrate digestion, particularly *α*-glucosidase and α-amylase, which play a key role in blood glucose regulation, and inhibition of these enzymes delays the hydrolysis of dietary starch in the digestive system, lowering blood glucose levels, slowing glucose metabolism, and delaying glucose absorption (62, 63). Structural analyses indicate that *α*-amylase activity requires calcium ions to maintain structural integrity and is activated by chloride ions, and similarly, α-glucosidase operates via a Koshland double displacement reaction mechanism (64). At present, plant-active peptides with *α*-glucosidase inhibitory activity in legumes, cereals and nuts have been studied, and the inhibition effect of different enzymatically hydrolysed protein peptides α-glucosidase has been found to be significantly different. Compared with commonly used enzymes including pepsin, trypsin, neutral protease, acid protease, bromelain, and flavour protease etc., alkaline protease, as an endonuclease with broad specificity, the enzymatically digested plant protein peptides have the strongest inhibitory effect on *α*-glucosidase, therefore, alkaline proteases are widely used to hydrolyze a variety of plant proteins (65, 66).

Soy protein peptide peptides prepared with alkaline protease (AP) exhibited the highest *α*-glucosidase inhibitory activity compared to peptides prepared by papain and trypsin digestion. Three novel *α*-glucosidase inhibitory peptides identified in soy protein peptides, LLPLPVLK, SWLRL and WLRL, had IC 50 s of 237.43 ± 0.52, 182.05 ± 0.74 and 162.29 ± 0.74 μmol/L, respectively (67).Two novel peptides (WH and WS) with strong *α*-glucosidase inhibitory activity were isolated from hydrolysate of almond oil processing residues. Peptide WH was relatively stable in simulated gastrointestinal digestion and was able to maintain the IC 50 value for α-glucosidase inhibition (17.03 ± 0.05 μmol/L), whereas WS significantly increased the IC 50 value after simulated digestion (24.71 ± 0.02 μmol/L to 44.63 ± 0.03 μmol/L) (68). The walnut protein product-derived peptide LPLLR, hydrolysed by alkaline protease, exhibited *in vitro* inhibitory activity against alpha-amylase and attenuated insulin resistance in HepG2 cells. Currently, it has become increasingly common to screen potentially active peptides by predicting the binding mode and affinity of peptides and *α*-glucosidase through molecular modelling techniques. Deng et al. (69) identified three new potentially active peptides, RWPFFAFM (1101.32 Da), AAGRLPGY (803.91 Da) and VVRDFHNA (957.04 Da), from mulberry leaves by molecular docking and molecular dynamics simulation. *In vitro* validation of RWPFFAFM and AAGRLPGY showed good IC50 values of *α*-glucosidase inhibition (1.299 mM and 1.319 mM). In addition, studies have shown that low molecular weight peptides have better stability and higher bioavailability, resulting in better biological functions *in vivo*. Kiwi enzymes hydrolyse wheat gluten proteins, where the smallest Mw fraction (< 1 kDa) of wheat alcohol soluble protein peptides showed the highest inhibitory capacity against *α*-glucosidase (18.4 ± 0.7%) and α-amylase (53.3 ± 1.9%) (70). According to previous studies, peptides with strong α-glucosidase inhibitory activity are short peptides with a relative molecular weight of less than 1 kDa, this is because lower molecular weight peptides can access and bind to the active site of α-glucosidase (71). Currently, acarbose is used as a glucosidase inhibitor, but long-term use of this drug can cause gastrointestinal side effects. Therefore, it is important to develop healthy, safe and efficient natural glucokinase inhibitors.

### 3.2 As an insulin secretion promoter and blood glucose regulator inhibitor inhibitor to regulate diabetic cognitive impairment

Biopeptides play a crucial role in enhancing insulin secretion, which is a central component of glucose regulation mediated by the pancreas through insulin and glucagon release. Enteric proinsulin hormones such as glucagon-like peptide-1 (GLP-1) and glucose-dependent insulinotropic polypeptide (GIP), GLP-1 promotes insulin production, reduces appetite, and maintains pancreatic *β*-cell health; whereas GIP enhances insulin secretion and affects fat metabolism and *β*-cell proliferation (72, 73). Rapid degradation of GLP-1 and GIP (DPP-IV) enzymes by dipeptidyl peptidase-IV emphasises the importance of DPP-IV inhibition. This inhibition extends the activity of these hormones, improves glucose control, maintains *β*-cell function, and ensures sustained postprandial insulin release for effective blood glucose reduction (74). Soymorphin-Soymorphin-5 (YPFVV), a β-opioid agonist peptide derived from the soybean β-glycin β-subunit, inhibits hyperglycemia in KKAy mice without increasing plasma insulin levels while decreasing plasma and hepatic triglyceride (TG) levels and liver weight, and promotes plasma adiponectin concentration and adiponectin receptor subtype AdipoR2 mRNA expression in the liver, PPAR in the liver after oral administration of soymorphin-5 The mRNA expression of *γ* and its target genes was also increased, effectively improving glucose and lipid metabolism in KKAy mice, a type 2 diabetes model animal (75). *In vitro* studies have shown that intraduodenal instillation of soy protein hydrolysate (SPH) in weaned piglets promotes the release of anorexigenic hormones such as peptide YY (PYY) and glucose-dependent insulinotropic polypeptide (GIP), stimulates insulin production in pancreatic islet cells by elevating the level of GLP-1 to inhibit short-term feed intake and triggers the secretion of cholecystokinin (CCK) in the liver through activation of the CaSR and the intracellular Ca^2+^/TRPM 5 pathway to reduce the appetite of pigs (76). Peptides RRDY and RL identified from yam diosgenin were used in oral glucose tolerance test (OGTT) on normal ICR mice, and peptide RRDY reduced DPP-IV activity controlling blood glucose levels in normal mice (77). The use of a mixture of corn and wheat peptides can weaken the autoimmune process of pancreatic inflammation, reduce the degree of infiltration of *β* cells, improve the function of pancreatic β cells, treat and prevent the development of type 1 diabetes, reduce the incidence of diabetes (78). In most studies, *in vivo* or *in vitro* experiments are often used to investigate the physiological effects of peptides. However, in order to screen potential bioactive peptides more conveniently in the face of large and complex protein resources, researchers usually perform active peptide screening based on computer-analyzed molecular docking or quantitative conformational-activity relationship (QSAR) modelling indicative of the relationship between identified or known peptides and target proteins (79). R. Han et al. (80) reported that the application of Peptide Ranker web server and Pepsite 2 software confirmed that oilseed protein is a potentially important source of DPP-IV inhibitory peptides. Mudgil et al. (81) hydrolysed quinoa protein using food-grade enzymes, identified 136 peptides, 35 of which were predicted as potentially bioactive peptides (BAPs) based on the Peptide Ranker score, and have high potential for inhibition of DPP-IV, AG, and ACE, DPP-IV inhibition is a key target in the treatment of T2DM, and DPP-IV inhibitors were among the first oral hypoglycaemic agents prospectively designed as glucose-lowering agents. To date, more than a dozen DPP-IV inhibiting drugs have been developed and marketed around the world, which are classified as gliptins (82). However, these synthetic DPP-IV inhibitors have been reported to have a number of adverse effects, such as gastrointestinal adverse reactions, allergic reactions, skin-related side effects, and musculoskeletal disorders.

### 3.3 Promote glucose uptake and cellular metabolism to regulate diabetic cognitive impairment

A key aspect of diabetes management is enhancing glucose uptake and regulating cellular metabolism. Glucose is the only source of energy in the brain because the brain cannot use fat or protein as alternative energy sources. In the brain, neurons have the highest energy requirements, but they cannot produce and store glucose, and therefore require a continuous supply of glucose to neurons via sodium-dependent glucose cotransporter family proteins (SGLT1 and SGLT2) and sodium-independent glucose transporter proteins (GLUT) across the BBB (83–85). In addition, glucose plays an important role in hippocampus-dependent learning and memory by upregulating the neurotrophic factors fibroblast growth factor-1 (FGF-1) and brain-derived neurotrophic factor (BDNF) through activation of the Akt signalling pathway (86). Neurons provide ATP mainly through mitochondrial oxidative phosphorylation (OXPHOS), glycolysis, and patients with cognitive impairment are associated with abnormalities in cerebral glucose utilisation as well as glycolysis and OXPHOS metabolism. Autopsy studies in patients with neurodegenerative diseases have shown significant reductions in GLUT1 and GLUT3 in brain regions that are closely associated with the pathology of cognitive impairment (87). Clinical studies using fluorodeoxyglucose FDG and positron emission tomography (FDG-PET) imaging studies in subjects with AD have shown reduced glucose transport and glucose metabolism in the areas most affected by AD (88). Meanwhile, reduced expression and translocation of the high-volume insulin-sensitive glucose transporter protein GLUT4 has been found in hippocampal neurons of patients with T2DM, leading to reduced neuronal glucose and ultimately cognitive dysfunction (89).

Biopeptides derived from medicinal plants have been shown to activate specific signalling pathways, in particular the AMPK pathway, a serine/threonine kinase that is activated when cellular energy levels are low, and which signals to stimulate glucose uptake, fatty acid oxidation in adipose (and other) tissues (90), glucose transporter protein (GLUT)4 translocation and mitochondrial biosynthesis, while inhibiting protein and glycogen synthesis and improving insulin sensitivity and glucose homeostasis. GLUTs are proteins that aid in the transport of glucose to various tissues where it is efficiently used as an energy source (91). Among the GLUTs, GLUT4 is considered the major insulin-regulated glucose transporter protein and is essential for glucose uptake. Soya globulin peptides (IAVPGEVA, IAVPTGVA and LPYP) from soybean activate GLUT1 and GLUT4 by stimulating the Akt and AMPK pathways in HepG2 cells and consequently promote energy metabolism (92). Pea oligopeptides have also been found to have great potential to reverse the metabolic abnormalities associated with type 2 diabetes, Y. Zhu et al. (93) demonstrated that four polypeptides, VLP, LLP, LL and LL, derived from pea significantly regulated glucose metabolism and exerted an antioxidant effect in IR-HepG2 cells. Among them, LLP, VA and LL promote the expression of GLUT2 genes and proteins, while VLP and LL inhibit p38 MAPK phosphorylation, improve glucose tolerance, restore pancreatic function and enhance insulin signalling. The lupine seed protein congly-g stimulates the specific pathway PKC/Flotillin-2/caveolin-3/Cbl to activate glucose homeostasis and increase glucose transport (94). The active peptides HTL, FLSSTEAQQSY and TLVNPDGRDSY were isolated and characterised from mung bean, and these peptides promote translocation of (GLUT4) to the plasma membrane. The tripeptide HTL promotes glucose uptake through activation of adenosine monophosphate-activated protein kinase, whereas the oligopeptides FLSSTEAQQSY and TLVNPDGRDSY promote glucose uptake through activation of the PI3K/Akt pathway, which is facilitated in L6 myotubes (95). Black bean-derived peptides (AKSPLF, ATNPLF, FEELN and LSVSVL) effectively reduced glucose uptake in Caco-2 cells, and molecular docking studies showed that these peptides strongly interacted with the glucose transport proteins SGLT-1 and GLUT-2. In a hyperglycaemic rat model, black bean hydrolysed protein isolate (HPI) reduced postprandial blood glucose in a dose-dependent manner in rats (Luis (96)). Taken together, these findings highlight the role of dietary biopeptides in regulating glucose metabolism, enhancing insulin sensitivity, and providing promising avenues for the regulation of diabetes.

### 3.4 Regulation-related inflammation affects cognitive dysfunction in diabetes

Phyto-derived bioactive peptides play a potential therapeutic role as anti-inflammatory and neuroprotective agents, addressing excitotoxicity, which contributes to improved neuronal viability (97, 98). These approaches can be combined with improving targets related to glucose metabolism to collectively improve cognitive dysfunction in diabetes. Currently, the use of rational *in vivo* animal models and *in vitro* experimental assays is important in evaluating the effects of bioactive peptide use (99), however, it is difficult to take into account bioavailability-related factors such as gastrointestinal digestibility and peptide utilisation for the assessment of learning memory capacity using neuronal cells alone. The Morris water maze test and passive avoidance test are usually adopted to evaluate the bioavailability of peptides at the animal level (100), while *in vitro* experiments are used to explore the molecular mechanisms associated with the action of active peptides (101). Multiple implant-derived bioactive peptides were found to significantly improve neuroinflammation and effectively delay the development of cognitive dysfunction in the diet.

Walnut proteolytic digests are a potential source of bioactive peptides with improved cognitive function. S. Wang et al. (102) demonstrated that walnut protein hydrolysate (WPH) and its low molecular weight grades (WPHL) could attenuate LPS-induced memory deficits by normalising inflammatory responses and oxidative stress in the brain. Recent studies have shown that soy protein hydrolysates and their active peptides have an ameliorative effect on memory disorders. Oral administration of soy peptide (SP) has been reported to attenuate age-related cognitive decline in learning and memory in a mouse model of accelerated ageing (SAM), with increased expression of neurotrophic factors, such as BDNF and NT-3, observed in the brains of SP-fed mice at both the mRNA and protein levels (103). Improvement in cognitive function may be attributed to upregulation of brain-derived neurotrophic factor (BDNF) and neurotrophic factor-3 (NT-3) levels by cAMP response element binding protein (CREB) activation in the brain. BDNF and NT-3 are members of a family of neurotrophic factors that support neuronal development and survival and are involved in memory formation and neurogenesis. BDNF deficiency leads to age-related cognitive impairment (104, 105).Montserrat-de la Paz et al. (106) showed that the blue lupin peptide GPETAFLR can exhibit anti-inflammatory properties by reducing TNF-*α* expression and inhibiting inflammatory cytokines (e.g., IL-1β, IL-6 and IL-10). In addition, it effectively inhibited nitric oxide production in microglia (BV-2 cells) and hindered the expression of pro-inflammatory genes in microglial cells of mice on a high-fat diet, attributes that suggest that blue lupin has the ability to attenuate inflammation (107, 108), process that is at the core of neurodegenerative diseases, and also demonstrated to have an enhanced potential for cognitive function. Wattanathorn et al. (109) found that cashew protein hydrolysate inhibited the production of pro-inflammatory cytokines in the brain of rats with arterial occlusion-induced cerebral is chaemia, and by modulating the function of lipid metabolism, its modulation of serum cholesterol, TG, and LDL, and elevation of HDL, greatly improved spatial memory in rats. Oat peptides (DF-10), (HL-8) and (RW-9) have been shown to improve behavioural performance, reduce AChE activity, attenuate oxidative stress, and decrease the levels of inflammatory cytokines including TNF-*α*, IL-6, and IL-1β in the brain of zebrafish exposed to scopolamine (110). Another study showed that the amino acid sequence and composition of peptides have a significant effect on neuroprotection and that the antioxidant and anti-inflammatory effects of peptides are attributed to the presence of hydrophobic aromatic amino acids and essential amino acids implicated in neuroprotection, which can be involved in a number of cellular processes, transported via the BBB, and modulation of neuronal memory (111). The lipolysis-stimulating peptide VHVV obtained from flavoured enzyme-soy protein (SPI) hydrolysate (F-SPIH) is composed of two essential amino acids, valine and histidine, where valine is involved in many cellular processes, such as lipolysis, lipogenesis, glucose transport, and intestinal barrier glucose metabolism, and histidine regulates neurogenesis, astrocytes, and BBB integrity (112).

### 3.5 Regulating oxidative stress affects diabetic cognitive impairment

Most of the plant antioxidant peptides consist of 2–20 amino acids. It can effectively scavenge excessive reactive oxygen free radicals in the body, protect the normal structure and function of cells and mitochondria, and prevent the occurrence of lipid peroxidation, thus playing a role in preventing cognitive dysfunction in diabetes (113). In recent years, plant-derived antioxidant peptides have attracted much attention due to their significant neuroprotective potential and their ability to cross the gastrointestinal barrier or the BBB to reach their target sites associated with their molecular mode of action (114). N. Li et al. (115) found that wheat germ peptides have an important role in the endogenous antioxidant system by enhancing the activities of antioxidant enzymes, such as GSHPx, superoxide dismutase (SOD), and catalase (CAT), while decreasing the production of malondialdehyde (MDA), in order to protect the PC12 cells from H_2_O_2_-induced oxidative stress. AREGETVVPG reduces intracellular reactive oxygen species (ROS) production, inhibits phosphorylation of PKCζ, AKT, and Erk 1/2, and inhibits Nox 4 protein expression to protect hyperglucose-induced vascular smooth muscle cells (VSMC) oxidative stress (116). Walnut peptides have similar antioxidant properties. In a previous study, walnut peptides demonstrated a protective effect against H_2_O_2_ and Aβ-induced cellular damage, which was accompanied by a decrease in lipid peroxidation and an increase in antioxidant enzyme activity in rat PC12 and SH-SY5Y cell lines, as well as in primary cultured cortical neurons (117). Notably, compounding walnut peptide with ginseng saponin to feed senescence-accelerated mice (SAM) revealed that walnut peptide significantly increased the serum levels of antioxidant enzymes and reduced Aβ and p-tau in the hippocampus through activation of the brain-derived neurotrophic factor (BDNF)/TrkB-dependent PI3K/Akt signalling pathway, significantly improving the memory of rats with neurodegenerative disease capacity (118). H. Chen et al. (119) identified 77 peptides from the antioxidant fraction of defatted walnut meal hydrolysate (DWMH) that exhibited relatively strong hydroxyl scavenging and oxygen radical uptake. In an animal model of D-galactose-induced neurodegeneration, DWMH eliminates spatial learning memory deficits in the Morris water maze experiment and the dark/light avoidance experiment in mice. Among them, WSREEQEREE and ADIYTEEAGR significantly ameliorated H_2_O_2_-induced oxidative damage in PC12 cells. Feng et al. (120) smulated gastrointestinal digestion of SGGY tetrapeptide obtained from DWMH and exhibited high radical scavenging activity in both 2,2′-azino-bis (3-ethylbenzothiazolino-6-sulfonic acid; ABTS) and oxygen radical absorbance capacity (ORAC) assays, which protects neuroblastoma (SH-SY5Y) cells from H_2_O_2_-induced oxidative damage. In addition, Zheng et al. (121) found that tripeptides from defatted peanut (*Arachis hypogaea*) meal hydrolysate (DPMH) with Tyr-Gly-Ser (YGS) structure had a strong free radical absorbance capacity (ORAC), preventing linoleic acid peroxidation, and H_2_O_2_ induced oxidative damage to PC12 cells had a protective effect. Its antioxidant activity may be mediated through the mechanism of transferring hydrogen atoms and the presence of Tyr at the N-terminus, which constitutes a hydrogen donor. Notably, the isolation of PGCPST from peanut protein hydrolysate not only exhibited desirable antioxidant capacity, effectively increased cell viability and reduced apoptosis in 6-OHDA-induced PC12, but also exerted neuroprotective effects through sphingolipid metabolism-related pathways (122).

### 3.6 Regulation of intestinal dysbiosis affects diabetic cognitive impairment

The intestinal flora, a complex community of microorganisms present in our gastrointestinal ecosystem, is an important protective barrier that maintains the integrity and structure of the intestinal layer, fights against harmful pathogens, and modulates host immunity, and imbalances in the intestinal flora can lead to impaired intestinal barrier function (123). Gut microbe-derived metabolites such as ROS and lipopolysaccharides can leak into the body circulation via a bidirectional microbe-gut-brain axis communication system, and these neurotoxin releases from the gut microbiota can elicit an inflammatory response and cross the blood–brain barrier to modulate neuronal activity, and neuroinflammation has been implicated in the pathogenesis of diabetes mellitus and its associated cognitive dysfunction (124). Y. Zhang et al. (125) used 16S rRNA gene sequencing to examine the composition of the intestinal flora in 154 patients with type 2 diabetes mellitus, 73 of whom had normal cognitive function and 81 of whom had impaired cognitive function. Tenericutes abundance is lower in patients with cognitive impairment. Comparisons at the genus level showed decreased abundance of Bifidobacterium and unranked-RF 39 and increased abundance of Digestive Cocci and unranked Leuconostocaceae in cognitively impaired patients with T2DM. In addition, the relative abundance of Veronococcus and Katococcus was reduced in cognitively impaired subjects, and the relative abundance of each of the seven subfunctions was significantly altered in the cognitively impaired group. Alterations in the gut microbiome have also been demonstrated in preclinical studies. H. Gao et al. (126) conducted a study on advanced type 1 diabetic (AST1D) rats with cognitive deficits and age-matched controls (AMC) to investigate the diversity of microbial populations. Microbiome alterations were found to be significant in ASTID rats. Relatively high abundance of Mycobacterium anisopliae and lower abundance of Mycobacterium anisopliae were observed in AST1D rats. Energy metabolism, which is critical for each organism, was significantly reduced in AST1D rats, especially in serum and hippocampus. Numerous findings have demonstrated that peptides can alleviate neuroinflammation and thereby ameliorate cognitive impairment by modulating the levels of reactive oxygen species in the gut and the composition of the intestinal flora, and that peptides with small molecular weights have higher antioxidant capacity compared to natural proteins and can be absorbed directly in the gut (126). Soy protein hydrolysates with molecular weight < 3 kDa not only attenuate the accumulation of reactive oxygen species ROS in H_2_O_2_-damaged human intestinal Caco-2 cells, but also inhibit lipid peroxidation and activate the cellular antioxidant defence system to protect the Caco-2 cells from oxidative stress, thereby maintaining the integrity of human intestinal mucosa (127). In addition, peptides can improve the composition of microbial communities by reducing the abundance of pathogenic organisms and protect the intestinal and mucosal immune systems, thereby exerting functional properties in neurodegenerative diseases. T2DM mice treated with ginseng polypeptide (GP) on a high-sugar and high-fat diet (including streptozotocin (STZ)) showed that GP could play a hypoglycemic role by restoring the SCFA-producing microbiota in the intestine, and enriched the microflora closely related to lipid metabolism, oxidative stress and inflammatory response, such as desalted bacilli, Bifidobacterium spp., and bacteroides (128). Targeting the insulin signalling pathway may provide new strategies for the prevention and treatment of cognitive impairment. Probiotic fermentation technology (PFT) has been reported to improve insulin signalling by modulating the gut microbiota, upregulating insulin receptor expression and activating PI3K/Akt signalling, followed by inhibition of GSK-3β and mTOR signalling, which leads to the downregulation of over-phosphorylated tau proteins in order to halt the development of memory and cognitive impairment (129). M. Wang et al. (130) reported that oral administration of PKNW enhanced learning and memory in APP/PS1 transgenic mice by reducing hippocampal Aβ plaque accumulation, and furthermore, PKNW treatment increased the relative abundance of the thick-walled phylum, whereas it decreased the relative abundance of the anamorphic phylum and the warty microphytobacterial phylum. Improved bidirectional communication between the gut-brain axis including the central and enteric nervous systems links the cognitive centres of the brain to peripheral gut function. Alterations in the gut microbiome are strongly associated with cognitive function. Further studies on interactions, metabolism and mechanisms of action are needed to develop effective neuroprotective agents (131). Given that peptides play an effective role in improving the composition of the gut microbiota, maintaining homeostasis of the gut microbiota prevents or delays the onset of neurodegenerative diseases. Thus, peptides may serve as a new research direction for future prevention and treatment of neurodegenerative diseases through microbial targeting to reduce the dependence on synthetic drugs with severe side effects (Table 1).

**Table 1:** Antidiabetic effects of peptides and protein hydrolysates identified from plants and their cognitive dysfunction.

Source	Protein hydrolysate	Peptide(s) identified	Mechanism of action	Reference
Soybean seeds	Soy protein hydrolysate	WSREEQEREE	Alleviate age-related cognitive decline in learning and memory in accelerated ageing mouse (SAM) models, upregulate increased expression of neurotrophic factors such as BDNF and NT-3 at the mRNA and protein levels.	(132)
	Soybean hydrolysate		Inhibition of lipid peroxidation and stimulation of antioxidant enzyme activity protect Caco-2 cells from H_2_O_2_-induced oxidative stress and maintain human intestinal mucosal integrity	(127)
	soy protein hydrolysates	LLPLPVLK	α-glucosidase inhibitors in vitro	(67)
		SWLRL		
		WLRL		
Almond (Armeniaca sibirica) oil		WH	α-glucosidase inhibitors in vitro	(68)
		WS		
Mulberry Leaves		RWPFFAFM	α-glucosidase inhibitors in vitro	(69)
		AAGRLPGY		
Wheat gliadin peptide		WGLYH	α-Amylase inhibitors in vitro	(70)
			α-glucosidase inhibitors in vitro	
Yam Dioscorin		RRDY	DPP-IV inhibitors in vitro	(77)
		RL		
Soy *β* - Glycin		YPFVV	Inhibits hyperglycemia in KKAy mice, decreases plasma and hepatic triglyceride (TG) levels, increases plasma adiponectin concentrations, participates in the stimulation of peroxisome proliferator-activated receptor (PPAR) β pathways and fatty acid *β*-oxidation in vivo.	(75)
Oilseeds	Oilseed protein hydrolysate		DPP-IV inhibitors in vitro	(80)
Quinoa	Quinoa protein hydrolysate		DPP-IV inhibitors *in vitro*	(81)
Pea seeds		VLP	Highly effective in stimulating glucose metabolism and alleviating oxidative stress in IR-HepG 2 cells via IRS-1/PI3K/AKT and p38 MAPK signalling pathways.	(93)
		LLP		
		VA		
		LL		
Lupin seed Protein conglutin-γ			Stimulation of specific pathways PKC/Flotillin-2/caveolin-3/CBL activates glucose homeostasis and increases glucose transport.	(94)
Mung bean seeds		HTL	Activation of Jak 2- and PI 3 K/Akt- pathways induces glucose uptake, activates leptin receptor/Jak 2/AMPK and PHT-1/AMPK signalling pathways.	(95)
		FLSSTEAQQSY		
		TLVNPDGRDSY		
Black bean seeds		AKSPLF	Blocks glucose transporters GLUT 2 and SGLT 1, thereby decreasing glucose uptake in Caco-2 cells.	(133)
		ATNPLF、		
		FEELN		
		LSVSVL		
Walnut		PPKNW	To ameliorate the intestinal dysbiosis of APP/PS1 transgenic mice by increasing the relative abundance of Firmicutes and decreasing the phylum Proteobacteria and verrucous microbacteria	(130)
	Walnut protease hydrolysate		Normalises the inflammatory response and oxidative stress in the brain, thereby alleviating LPS-induced memory impairment	(102)
	Defatted walnut hydrolysate	SGGY	Activates intracellular antioxidant defences to protect PC 12 cells from glutamate-induced apoptosis (superoxide dismutase (SOD) and glutathione peroxidase (GSH-Px)), inhibits ROS production, Ca ~ (2+) influx, and reduction of mitochondrial membrane potential (MMP) and apoptosis-related proteins Bax and Bcl-2 by Kelch-like ECH-associated protein 1 (Keap 1) inhibition.	(117)
		WSREEQEREE		(132)
		ADIYTEEAGR		
Blue lupine seeds		GPETAFLR	Reduces TNF-α expression and inhibits the release of inflammatory cytokines such as IL-1β, IL-6, and IL-10, while inhibiting nitric oxide production in microglia (BV-2 cells).	(106)
Cashew	Cashew protein hydrolysate		It inhibits the production of pro-inflammatory cytokines, regulates serum cholesterol, TG and LDL, increases HDL, and improves spatial memory in rats	(109)
Oats	Oat protein hydrolysate	DFVADHPFLF	Reduces MDA levels in zebrafish brain, enhances AChE activity and jiangdi inflammatory cytokine levels, and reduces oxidative stress by regulating Nrf 2-keap 1/HO-1 gene expression.	(110)
		HGQNFPIL		
		RDFPITWPW		
Wheat		AREGETVVPG	Reduces intracellular reactive oxygen species (ROS) production, inhibits phosphorylation of PKCζ, AKT and Erk 1/2, and inhibits Nox 4 protein expression.	(116)
Peanut seeds	Defatted peanut hydrolysate	YGS	Protects linoleic acid from peroxidation and protects PC12 cells from hydrogen peroxide-induced oxidative damage.	(121)
	Peanut protein hydrolysate	PGCPST	Protection of 6-OHDA-induced apoptosis in PC12 by sphingolipid metabolism-related pathways, validated in vitro with neuroprotective effects	(122)
Ginseng			GP regulates glucose and lipid metabolism disorders, IR, oxidative stress, and anti-inflammatory responses; It improves intestinal microbiota dysbiosis, and regulates glycogen synthesis, gluconeogenesis, and glucose transport by activating the IRS-1/PI3K/Akt and AMPK signalling pathways, and improves IR by inhibiting the mitochondrial apoptosis signalling pathway	(128)

## 4 Conclusion

Over the years, diabetic cognitive dysfunction has been extensively studied as a complication and comorbidity of diabetes, and although the specific pathogenesis is still unclear, a large number of studies have found that diabetic cognitive dysfunction and neurodegenerative diseases (e.g., Alzheimer’s disease) share common pathophysiological characteristics. With the progress of research, it has been found that chemical synthetic drugs that regulate diabetes may improve cognitive dysfunction and prevent the occurrence of dementia to a certain extent, however, these synthetic drugs have certain side effects that may bring inconvenience to patients’ lives. In contrast, phytogenic bioactive peptides have a wide range of sources, low molecular weight, high activity and specificity, easy absorption, high safety and low toxicity, and have broad application prospects in the prevention and treatment of various diseases. This article reviews the relevant mechanisms affecting the onset of cognitive dysfunction in diabetic patients, including oxidative stress, neuroinflammation, tau hyperphosphorylation, and amyloid precipitation. The development of these symptoms involves dysfunction of insulin signalling or synthesis, impaired glucose transporters. Here, we discuss potentially active peptides with antidiabetic properties that inhibit *α*-amylase, α-glucosidase, DPP-IV, and have inhibitory oxidative stress, apoptosis, and inflammatory and neuroprotective peptides isolated from plant sources. At present, additional work is needed to verify the efficacy and safety of phytogenic bioactive peptides in anti-diabetic and neuroprotective, so as to escort the further development of foods and drugs with the ability to prevent diabetic cognitive dysfunction.
